# Co-Designing Digital Health Intervention for Monitoring Medication and Consultation Among Transgender People in Underserved Communities: Collaborative Approach

**DOI:** 10.2196/45826

**Published:** 2024-09-12

**Authors:** Emmanuel Oluwatosin Oluokun, Festus Fatai Adedoyin, Huseyin Dogan, Nan Jiang

**Affiliations:** 1 Department of Computing and Informatics Faculty of Science and Technology Bournemouth University Poole, Dorset United Kingdom

**Keywords:** digital health, HIV/AIDS medication, data-driven health care, ART, transgender, LGTBQI+, interactive management

## Abstract

**Background:**

In many parts of the world, men who have sex with men and transgender individuals face criminalization and discrimination. As a result, they are less likely to seek medical help, despite experiencing higher rates of HIV/AIDS, mental health issues, and other health problems. Reaching key populations (KPs) with essential testing, care, and treatment services can be challenging, as they often have a higher likelihood of contracting and spreading the virus. They have limited access to antiretroviral (ARV) therapy (ART) services, which means that KPs may continue to serve as reservoirs for new HIV infections if they do not receive effective HIV programming. This ongoing issue complicates efforts to control the epidemic. Therefore, modeling a digital health system to track ARV medication access and use is crucial. This paper advocates for the use of digital interventions to manage the health of KPs in underserved regions, using Nigeria as a case study.

**Objective:**

This study aims to assess digital health interventions for monitoring medication and consultations among transgender people in underserved communities. It also sought to determine whether a system exists that could support ART adherence in Nigeria. Additionally, the study evaluated design strategies to address privacy and confidentiality concerns, aiming to reduce nonadherence to ARV medications among KPs in Nigeria.

**Methods:**

A qualitative approach was adopted for this research, involving a thematic analysis of information collected from interviews with clinicians and other health practitioners who work directly with these communities, as well as from an interactive (virtual) workshop.

**Results:**

The findings from the thematic analysis indicate a need to increase attendance at ART therapy sessions through the implementation of an intensive care web app. Unlike previous solutions, this study highlights the importance of incorporating a reminder feature that integrates with an in-app telemedicine consultancy platform. This platform would facilitate discussions about client challenges, such as adverse drug effects, counseling sessions with clinical psychologists, and the impact of identity discrimination on mental health. Other data-driven health needs identified in the study are unique drug request nodes, client-led viral load calculators, remote requests, and drug delivery features within the web app. Participants also emphasized the importance of monitoring medication compliance and incorporating user feedback mechanisms, such as ratings and encouragement symbols (eg, stars, checkmarks), to motivate adherence.

**Conclusions:**

The study concludes that technology-driven solutions could enhance ART adherence and reduce HIV transmission among transgender people. It also recommends that local governments and international organizations collaborate and invest in health management services that prioritize health needs over identity.

## Introduction

### Background

The HIV infection rate in Nigeria is the third highest in the world [1]. As of 2018, 1.9 million people in Nigeria were living with HIV/AIDS, according to the 2019 Nigeria National HIV/AIDS Indicator and Impact Survey [2]. HIV and AIDS are much more prevalent among incarcerated individuals, high-risk drug users, sex workers, transgender people, men who have sex with men (MSM), and people who inject drugs. Studies by the United Nations Office on Drugs and Crime found that 9% of people who inject drugs [3] and 2.8% of incarcerated individuals in Nigeria are living with HIV/AIDS [1]. These rates are significantly higher than the estimated 1.4% prevalence in the general population [1].

HIV/AIDS is undoubtedly a challenging condition, as it requires lifelong therapy and may lead to the emergence of new HIV-associated complications, as noted by Deeks et al [4]. Although combination antiretroviral (ARV) therapy (ART) can improve the health of patients infected with HIV, barriers to effective ARV treatment can negatively affect outcomes for those living with the disease [5]. HIV/AIDS is a highly stigmatized condition, and individuals with HIV/AIDS are more likely to be diagnosed with other health issues, such as poor mental health, psychiatric disorders, and substance use disorders. These additional, negatively perceived conditions can increase stigma and hinder adherence to care [6,7]. Therefore, it is crucial to explore the benefits of digital health interventions for monitoring medication and consultations among people living with HIV/AIDS.

It is crucial to provide key populations (KPs) with necessary testing, care, and treatment services, as they are at higher risk of contracting and spreading the virus. Unfortunately, these groups often have limited access to ART services. Without proper HIV programming, KPs may continue to serve as a reservoir for new infections, hindering efforts to contain the pandemic [8]. There is a need to expand knowledge on the use of digital health innovations in ART delivery. However, there is a lack of understanding regarding the ethical creation and use of these digital health solutions [9]. This study aims to cocreate a digital health solution to track ARV drug access among transgender people in underserved communities. It also seeks to determine if a system exists that can be utilized to support ARV adherence in Nigeria. The study also explored design strategies to address privacy and confidentiality concerns among KPs, aiming to reduce nonadherence to ARV medications in Nigeria.

This paper advocates for the role of digital interventions in managing the health of KPs in underserved regions, using Nigeria as a case study. The study makes several innovative contributions: first, it documents for the first time in the literature the challenges faced by health care professionals working with KPs in Nigeria; second, it introduces a trigger question on the role of digital health interventions through an interactive management (IM) approach; and third, it outlines the cocreation process with users in developing a digital solution, presenting this as an agenda for future research. The “Literature Review” section reviews the literature on ART and digital interventions, followed by the “Methods” section, which outlines the research approach and methods used in this study. The key findings (the “Results” section) and discussions (the “Discussion” section) are then presented, followed by vital recommendations and an agenda for future research in the “Conclusions” section.

### Literature Review

ART has transformed HIV infection from a fatal illness into a manageable chronic condition [10]. ART can also reduce viral load (ie, levels of HIV RNA) and the risk of secondary transmission, establishing a new preventive paradigm where effective scaling of therapy could contribute to the end of AIDS [11]. Iyun [12] found that the majority of the 35 million individuals testing positive for HIV live in resource-limited settings. In 2016, an estimated 17 million of them were receiving ART, up from 1.3 million in 2006. Goals for 2020 projected treatment for an additional 20 million patients [13]. Although this expansion of ART is one of the greatest public health achievements of our time, significant challenges remain. Patients require near-perfect adherence of at least 95% to maintain undetectable viral loads and support immune system activity, making adherence to ART a considerable challenge.

In Nigeria, HIV prevalence rates among lesbian, gay, bisexual, transgender, queer, and intersex (LGBTQI) communities and MSM are up to 19 times higher than those in other populations [14]. Analyzing these groups is particularly challenging due to the limited number of studies and the insufficient funding allocated to this community. In a study by Liu et al [15], only 45% of MSM reported good adherence to ART. Low adherence rates are attributed to various issues, including HIV stigma, social exclusion, limited access to health care programs, fear of seeking care or being denied it, depression, and insufficient information about drug interactions between hormonal therapy and ART [16]. A recent meta-analysis [17] revealed that only 77% of sub-Saharan Africans on ARV medications adhered to the recommended dosage schedule. Overall, there is limited information available on the adherence levels achieved by medical facilities providing regular ART services.

Individuals who use ARV medication erratically may experience side effects, minimal benefits, and fewer treatment options in the future. It is crucial that every patient understands this before starting treatment. If a patient discontinues ART entirely, they will quickly lose any gains in immunity due to the continued spread of the virus and destruction of CD4+ cells [18]. It is essential to emphasize to patients that ART is a lifelong commitment. Effective patient education and adherence assessments require significant time and effort, but this investment is worthwhile. Clinical negligence occurs when a prescription is provided only at the initial visit without adequate adherence counseling, a practice that unfortunately remains common [19].

Transportation issues, such as difficulties reaching the nearest medical facility, combined with fears of job loss from taking time off work, have made consistent adherence to ART challenging [20-23]. Additionally, several interview-based studies have identified patients’ attitudes toward ART as a factor contributing to the rejection of HIV/AIDS antiretroviral therapy (HAART) [24,25]. People with HIV/AIDS have expressed various concerns about HAART, including worries about side effects, the need for strict adherence, inconvenience and practical issues related to the regimen, mistrust of conventional medications, fears of long-term organ damage, and the belief that treatment is unnecessary in the absence of symptoms. Additionally, concerns about the impact of HAART on self-identity and the potential for treatment to reveal their HIV status contribute to these negative opinions [26,27]. These concerns highlight the need to explore digital health solutions.

The potential of digital interventions in ART has yet to be fully realized, partly because it is challenging to build a comprehensive body of knowledge to guide decisions about digital health solutions. Information and communications technology is utilized in digital health interventions to improve health outcomes [28]. Digital innovations can be categorized into noninternet technologies (eg, SMS text messages and phone calls) and internet-based technologies (eg, social media, mobile apps, and websites) [29]. Internet-based digital interventions allow users to publish content and share information on sensitive topics at any time and from any location, potentially minimizing the risk of unintended disclosure of private behaviors. The recent globalization of instant messaging platforms has laid the foundation for internet-based digital interventions. For example, a widely used instant messaging service, which includes nonsmartphone options, has 2 billion monthly active users across 180 countries [30]. SMS text messages and real-time medication monitoring through such platforms have been promoted to enhance ART adherence [31-34].

Correspondingly, the World Health Organization (WHO) and other agencies have recommended using digital technologies to deliver adherence interventions [35]. The rapid advancement of the technological landscape requires the continuous evolution and updating of digital interventions to improve adherence to ARV medications. These digital technologies can promote healthy behaviors; enhance treatment outcomes for chronic conditions such as diabetes, heart disease, and mental health issues; and provide remote access to effective treatments (eg, computerized cognitive behavioral therapy for patients with HIV) [36]. Digital interventions are often complex, involving multiple components and goals. These can include empowering users to learn more about their health, share experiences with others in similar situations, change perceptions and beliefs about health, evaluate and track health states or behaviors, adjust medication, identify health priorities, make informed treatment decisions, and enhance communication between patients and providers.

However, there have been reports of negative effects associated with digital health interventions. Research conducted in some parts of Africa revealed concerns about unintended HIV disclosure [37-40], while others anticipated stigma from SMS text message content that included terms such as “medication” and “HIV” [41,42]. Systematic evaluations [43,44] have also found that low-resource environments, poor internet connectivity, and the high cost of smartphones and their maintenance have contributed to the failure of digital health interventions. Logistics issues, such as frequent sharing of mobile phones among family members, intermittent electricity availability, and mobile phone malfunctions, were also highlighted [45]. These factors can complicate the adoption of digital innovations by clinic personnel and end users [46]. Given the recognized implementation challenges associated with digital innovations, further research is needed to identify and address barriers that may limit the acceptance of these interventions in practical settings.

## Methods

### Research Design

A qualitative approach was adopted for the research, utilizing thematic analysis of data collected from an IM workshop and an interview session. This approach was chosen because it facilitates in-depth probing and questioning of participants based on their responses, allowing both participants and researchers to explore underlying reasons and sentiments. The study aims to understand the perspectives of health professionals on how digital health care interventions can best enhance the monitoring of ARV medications among KPs. Interaction among focus group participants can often yield more insights than one-on-one interviews. Therefore, relevant data are collected through an IM workshop and a follow-up interview.

### Ethics Approval

This study was approved by Bournemouth University (approval number 44608). The authors understand that their data may be used in an anonymized form by research teams to support other ethically approved research projects in the future, including future publications, reports, or presentations.

### Population and Sample

Health care practitioners participating in the study included doctors, pharmacists, nurses, and clinical psychologists from Lagos State, Nigeria. Two sampling techniques were used: purposeful and convenience sampling. The purposeful sampling technique was used to select health care professionals working within the KP community. The convenience sampling technique was then used to select 1 doctor, 1 nurse, 1 pharmacist, and 1 clinical psychologist, with additional participants being social workers who work with the KP. The 2 sampling techniques were chosen because they help identify participants who are well-suited for the study [47,48]. Because of the extensive ethical clearance required, the study could not include all relevant participants. The Initiative for Equal Rights (TIERs) was selected as the venue for the IM workshop, while the interview was conducted via Zoom (Zoom Video Communications/Qumu Corporation) after securing participant agreements for both the workshop and interview. The study sample consisted of 13 participants in total, with all participating in the IM workshop and 7 participating in the interview.

### Instrument

Initially, an open-ended survey was planned, accompanied by an interview guide with 5 questions aligned with the study’s objectives. Instructions were provided on how to respond to the questions and an explanation was given about the features of digital health care interventions for monitoring ARV medications among KPs. This was done to ensure participants had a clear understanding of the study and how to provide their responses. The interview questions are crucial for gathering information on factors pertinent to the study’s objectives.

### The Procedure of Administration

A formal agreement was established with each contact person to schedule an interview following the IM workshop, conducted as a focused group discussion. The interviews lasted between 20 and 30 minutes, with responses recorded for transcription. Participants provided informed consent by signing agreement forms after carefully reviewing the participant information page, which included all relevant details about the project, as required by ethical standards. During the interview, 5 questions were posed, and 7 participants actively responded. Their answers were recorded, transcribed by a researcher (FFA), and reported accordingly.

### Trustworthiness

Trustworthiness is a critical consideration in research as it allows researchers to demonstrate the value of findings beyond typical qualitative research parameters [47]. In this study, trustworthiness is aimed at reinforcing the significance of the findings. To achieve this, a pilot study was conducted to assess the reliability of the data. Additionally, an interview was conducted with 1 pharmacist and 1 doctor, and their responses were transcribed and returned to them for verification to ensure the accuracy of the content.

### Interactive Management Session

**Figure 1:**
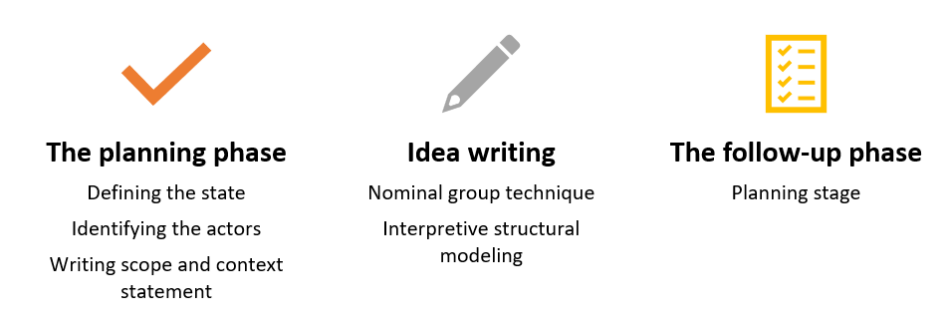
Interactive management flowchart.

Group work on complex issues is facilitated using computer assistance through IM [49]. IM can be viewed as a structured focus group method applied across various research fields, including cybersecurity. It uses human factor approaches [50] and requirements engineering [51] to support consensus decision-making through idea generation, structuring, and design. Typically, an IM session involves 8-12 participants who are knowledgeable about the topic and represent diverse viewpoints. The group usually convenes for 3-5 days, with follow-up sessions often occurring as needed. Before the working sessions, a detailed work plan is developed through collaboration between the workshop planner and an organizational representative. Participants are supported in generating, clarifying, and structuring concepts using well-established and effective approaches. The flowchart for conducting a successful IM session, as adopted for this research, is presented in Figure 1.

### The Planning Phase

#### Overview

The first and most crucial step in this phase is to understand the current situation. This involves defining the state, identifying the key actors, and drafting scope and context statements. These techniques help participants explore who is affected, what issues arise, and how they are impacted. By clearly defining the problem, participants can identify key questions that, when answered, could significantly expedite the development of effective solutions [49].

#### The Workshop Phase

Next is the workshop phase, where participants convene to address planning-related concerns and implement consensus decisions. The session focuses on 3 key concepts: context, content, and process [49]. The facilitator begins by guiding the discussions and providing context from the planning phase. The group then contextualizes the information through discussion and idea sharing. The facilitator manages the workshop flow to ensure discussions remain focused and participants make efficient use of their time.

### Idea Writing

Nominal group technique and interpretive structural modeling are the techniques covered in the IM workshop. Participants start by responding to a trigger question through idea writing. After exchanging written ideas with others, additional ideas are integrated. The compiled information is then categorized and presented to the group. Following this, the nominal group technique is applied, where participants generate further ideas based on the enhanced understanding of the issue gained from the initial idea writing. Additionally, the workshop facilitates the editing and clarification of problem statements. Participants assign priority ratings to each idea. The final stage of the workshop aims to convert idea statements into objectives, which are then used to construct an interpretive structural model to reveal connections between different aspects of the issue [52].

### The Follow-Up Phase

This stage marks the beginning of the solution implementation planning while putting the workshop’s goals into practice. If it is discovered during this phase that the problem was misunderstood or if new issues have emerged that were not previously considered, a new planning phase would be initiated [49].

### Thematic Analysis

**Figure 2:**
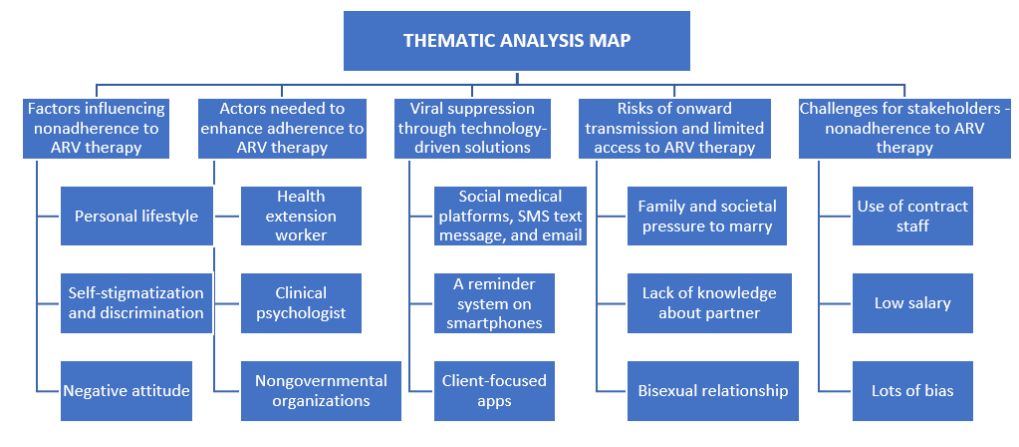
Thematic map capturing the core themes from the qualitative analysis. ARV: antiretroviral.

A thematic analysis was conducted on the core questions asked during interviews with key stakeholders working with KPs. The data were collected between July 2022 and August 2022. The process and core issues identified from the thematic analysis are presented in Figure 2 and are derived from the interview questions listed in Table 1.

**Table 1:** Interview questions and codes for creating themes from interview responses.

Question number	Interview questions	Codes for creating themes from interview responses
1	What are the key factors influencing nonadherence to ARV^a^ therapy among key populations?	Self-stigmatization, condition of health status, poverty, lack of sensitization and information, poor attitude, personal lifestyle, substance abuse, state of hopelessness, denial of diagnosis, cultural disposition, psychological distress, migration, logistics and financial constraint, privacy and confidentiality, the proximity of medical care facility, religious belief, hostile environment, drug side effect.
2	What category of actors do you consider relevant in the suppression of nonadherence to ARV therapy among key populations through technology-driven solutions?	International organizations, national organizations, NGOs^b^, health practitioners, religious organizations/leaders, and health extension workers are the key actors that are relevant in the suppression of nonadherence to ARV therapy.
3	How can we maintain viral suppression through technology-driven solutions among key populations?	Social media, SMS text messages, email, Google Maps, a reminder system on smartphones, embedded app software powered with AI^c^, client-focused apps, mobile apps, and interactive-driven systems are identified by the participants as a way of maintaining the viral suppression.
4	How do you consider the high risk of onward transmission and limited access to ARV therapy?	Key populations are not ready to share their problems with anyone, and at the same time, they engage in unprotected sex. The key populations also lack sufficient information to help prevent the spread of the virus, as well as awareness campaigns from key actors to address nonadherence to ARV therapy. Additionally, there is a lack of knowledge about one’s partner, societal and family pressure to marry at all costs, and self-denial of a diagnosis.
5	What are the challenges associated with working with the key stakeholders in the suppression of nonadherence to ARV therapy?	The workers working with the NGOs are contract staff; therefore, their salaries are not encouraging. Lack of collaboration between and among stakeholders as well as a lack of passion to work and lots of bias.

^a^ARV: antiretroviral.

^b^NGO: nongovernmental organization.

^c^AI: artificial intelligence.

## Results

### Factors Influencing Nonadherence to ART

**Figure 3:**
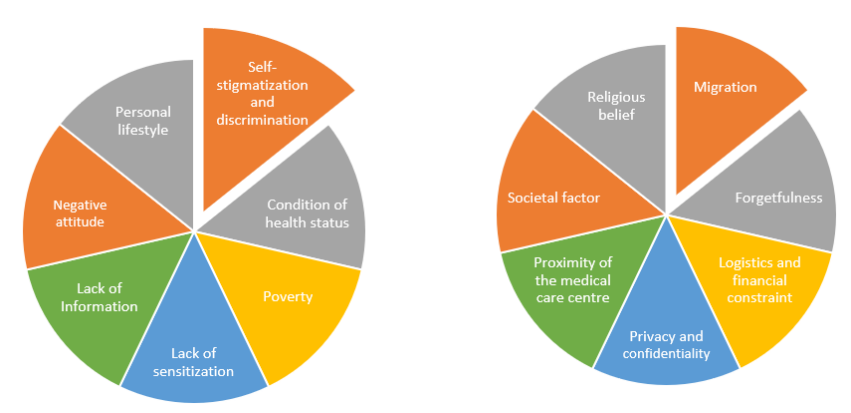
Thematic description of key factors influencing non-adherence to antiretroviral therapy.

The participants were asked about the key factors likely to influence nonadherence to ART among KPs. The findings revealed that factors such as self-stigmatization, health status, poverty, lack of sensitization and information, poor attitude, personal lifestyle, substance abuse, hopelessness, denial of diagnosis, cultural disposition, psychological distress, migration, logistical and financial constraints, privacy and confidentiality concerns, proximity to medical care facilities, religious beliefs, hostile environments, and drug side effects all contribute to nonadherence to ART. Participants provided various justifications for the factors influencing nonadherence to ART. However, self-stigmatization and discrimination, economic status, attitude, religious beliefs, and proximity to medical care facilities were ranked as the most significant factors, as shown in Figure 3.

### Actors Needed to Enhance Adherence to ART

**Figure 4:**
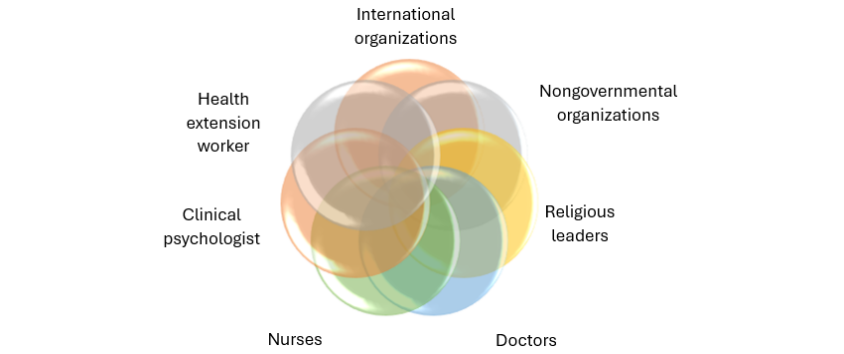
Thematic description of actors needed to enhance adherence to antiretroviral therapy.

Participants were asked to identify and categorize the key actors relevant to addressing nonadherence to ART among KPs. The findings indicated that international organizations, national organizations, nongovernmental organizations (NGOs), health practitioners, religious organizations/leaders, and health extension workers are crucial actors. Their categorization and ranking are as follows: NGOs, international organizations, national organizations, doctors, nurses, clinical psychologists, and religious organizations. This is illustrated in Figure 4, highlighting the significant influence of international organizations in financing health care interventions for KPs in Nigeria.

### Maintaining Viral Suppression Through Technology-Driven Solutions

**Figure 5:**
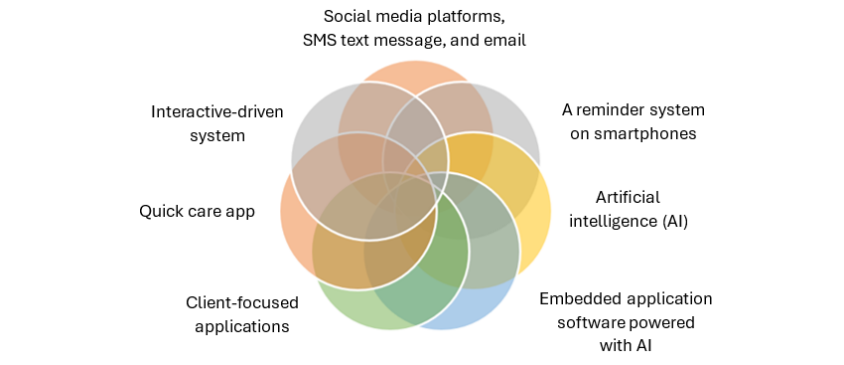
Thematic description of maintaining viral suppression through technology-driven solutions.

Participants were asked to suggest technological solutions for maintaining viral suppression among KPs. The results revealed that social media, SMS text messages, email, Google Maps, smartphone reminder systems, artificial intelligence–powered app software, client-focused apps, mobile apps, and interactive systems were identified as effective methods for maintaining viral suppression, as shown in Figure 5.

### Risks of Onward Transmission and Limited Access to ART

**Figure 6:**
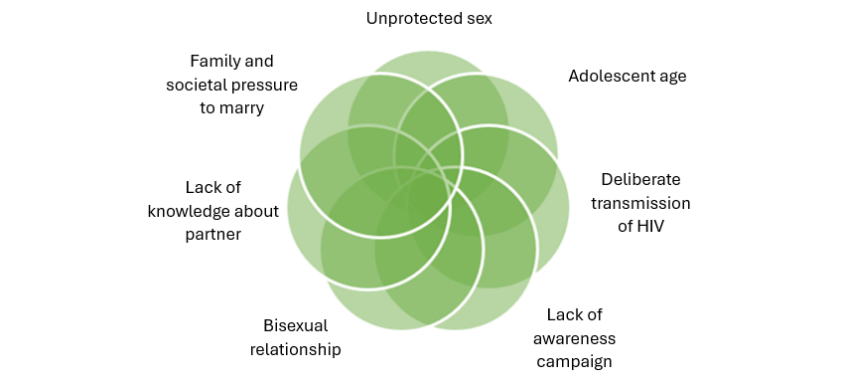
Thematic description of risks of onward transmission and limited access to antiretroviral therapy.

Participants were asked about factors contributing to the high risk of onward transmission and limited access to ART. The findings indicated that many KPs are unwilling to share their issues, engage in unprotected sex, lack information to prevent virus spread, experience insufficient awareness campaigns, engage in bisexual relationships, have limited knowledge about partners, face family and societal pressure to marry, and self-deny their diagnosis, as presented in Figure 6.

### Challenges With Stakeholders in the Suppression of Nonadherence to ART

**Figure 7:**
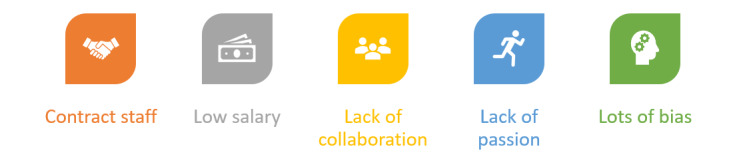
Thematic description of challenges with stakeholders.

Participants were asked to identify challenges in working with stakeholders to address nonadherence to ART. Findings, presented in Figure 7, highlight issues such as contract staff with inadequate salaries, lack of collaboration among stakeholders, lack of passion for the work, and prevalent biases.

### Results of the IM Workshop Session

The IM workshop (virtual) was facilitated by one of the authors (FFA) and took place in a conference room at Bournemouth University for the attendees’ convenience. A total of 8 participants were selected based on the following criteria: they must be a member of the social care community, volunteer within the LGBTQ community in Lagos, and have expertise in HIV/AIDS care provision. The exclusion criteria for the workshop included health care workers not involved in ARV medication provision and care. Of the 8 participants, 7 participated in the Zoom session. To facilitate idea writing, a trigger question was provided for participants to record their responses and exchange them. As a result of time constraints imposed by participants’ schedules, the phase of sharing ideas aloud began immediately. A computer paired with a projector was used to record and display the ideas.

The following are the trigger queries:

- What digital intervention can be used to improve ARV medication delivery and usage?
- How can we enhance viral suppression through technology-driven solutions?

Tables 2-5 present the ideas and responses generated from the trigger questions. These ideas were categorized as shown in Table 2, which outlines the different categories of responses. Table 3 highlights the significant concern for health care professionals regarding the development of a drug interaction monitoring system, as this was a major point of discussion among doctors, clinical psychologists, social workers, and other health care professionals working with KPs. The categories are ranked based on the number of ideas in each category. The IM session also identified the development of a web app, ideally named a KP Intensive Care App, and a medication use tracking system as crucial digital solutions or digital health care interventions.

**Table 2:** Results of idea generation.

Number	Idea
1	Development of the KP^a^ Intensive Care App that would enable easy access to and delivery of ARV^b^ medication.
2	A reminder feature to notify the client to take medication at scheduled times, and to remind them of upcoming appointments and drug pickups.
3	In-app telemedicine consultation to address client challenges, such as drug side effects and counseling needs.
4	Request for medication and viral load tests remotely, with delivery arranged through the app.
5	Pop-up messages to notify clients about potential drug interactions.
6	Avatar-guided animation videos demonstrating how to use medications, collect samples for viral load testing, and present samples within the app.
7	Section for booking appointments, including next drug pickup and doctor visits.
8	Monitor medication compliance and provide feedback for encouragement, such as stars or okay signs.
9	A feature that displays up-to-date tracking data, such as consultation records and viral load results, for both backend management and client benefits.
10	Development of a KP-friendly app, similar to a chatbot, to remind clients about medication schedules and clinic appointments.
11	Manufacturing medications or injectables with less frequent dosing to reduce pill burden.
12	Using dried blood spot collection for viral load testing can encourage clients, especially those with trypanophobia, to undergo viral load testing and adhere to their testing schedule.

^a^KP: key population.

^b^ARV: antiretroviral.

**Table 3:** Categorization of ideas.

Category	Ideas^a^	Ranking
KP^b^ Intensive Care App	1, 3, 10	2
Viral load collection system	4, 9	3
Medication use tracking device	2, 6, 8	2
Drug interaction monitoring system	5, 7, 11, 12	1

^a^Refers to the categorization of results of idea generation in Table 2.

^b^KP: key population.

Additionally, the nominal group technique was used to enable participants to prioritize the top 5 issues from the generated ideas, as shown in Table 4. Participants ranked each idea from 1 (most important) to 5 (least important). The results indicate that the development of a KP Intensive Care App, which facilitates easy access to ARV medication, provides a digital platform for monitoring medication compliance, and offers motivational feedback (such as stars or okay signs), is considered the most important. Moreover, within the web app, features such as reminders for drug use and appointments, management of drug requests, calculation of viral load, and remote ordering and delivery were also ranked as significant.

**Table 4:** Participant’s ranking of ideas.

Idea	Participant 1	Participant 2	Participant 3	Participant 4	Participant 5	Total score
1	1	3	1	5	1	11
2	—^a^	5	2	1	2	10
3	3	—	—	—	—	3
4	—	1	—	—	—	1
5	—	—	—	5	—	5
6	4	—	3	—	2	9
7	—	4	5	—	5	14
8	1	—	1	—	—	2
9	—	2	—	—	—	2
10	—	—	—	4	—	4
11	2	5	—	—	3	10
12	—	3	—	5	—	8

^a^Not available.

According to Table 4, ideas 7, 1, 2, 11, and 6 are ranked as the top 5 based on the total score.

**Figure 8:**
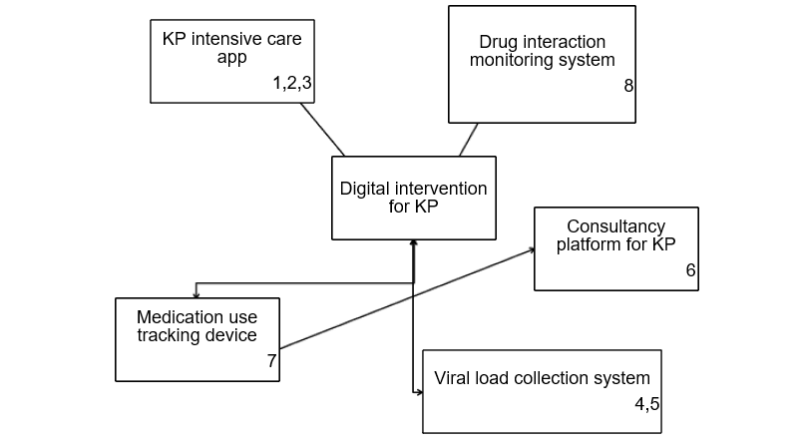
Interpretive structural model. KP: key population.

Table 5 displays the objective statements derived from the idea statements during the workshop. These objective statements were used to construct an interpretive structural model, as illustrated in Figure 8. The figure shows the grouping of similar objective statements, with numbers assigned to each box to reflect their categorization.

**Table 5:** Objective statements.

Number	Objective statements
1	Develop a KP^a^-focused intensive care app that provides easy access to and delivery of ARV^b^ medication.
2	Include unique reminder features to notify clients to take their medication at scheduled times, as well as to remind them of appointments and drug pickups.
3	Develop an in-app telemedicine consultancy platform for clinical psychologists to conduct private sessions with KPs.
4	Incorporate a drug request framework that depends on the accurate calculation of viral load from the app.
5	Provide pop-up messages to alert clients about potential drug interactions.
6	Include avatar-guided demonstrations and animation videos on how to use medications.
7	Establish a partnership with HIV/AIDS medication manufacturers to explore options for less frequent usage or dosage of medicines.
8	Develop a systematic and noninvasive data collection approach to encourage clients to participate in testing and adhere to their treatment regimen.

^a^KP: key population.

^b^ARV: antiretroviral.

## Discussion

### Summary

The study focused on utilizing digital health care interventions for the consultation and monitoring of ARV medication among transgender individuals in underserved regions. To achieve the study’s objectives, the following topics were investigated: factors influencing nonadherence to ART; key actors needed to improve adherence; methods to sustain viral suppression through technology-driven solutions; reasons for the high risk of onward transmission and limited access to ART; and challenges associated with collaborating with stakeholders to address nonadherence to viral treatment.

### Principal Findings

Indeed, the study’s findings revealed that several factors influence nonadherence to ART among MSM. These factors include self-stigmatization, health status, poverty, lack of awareness and information, poor attitude, denial of diagnosis, cultural disposition, psychological distress, migration, logistical and financial constraints, issues of privacy and confidentiality, proximity to medical facilities, and drug side effects. Indeed, these results align with a previously conducted study [15], which indicated that ART adherence was below average among MSM at risk for HIV infection in the United States. Several factors contribute to these low adherence rates, including HIV stigma, social exclusion, difficulties accessing health care programs, anxiety about seeking and potentially being denied care, depression, and a lack of knowledge about the interactions between hormonal therapy and various medications.

The findings also identified key players crucial for addressing nonadherence to ART, including international organizations, national organizations, NGOs, health professionals, religious leaders, and health extension workers. This outcome is supported by recent research conducted in South Africa, which concluded that a shortage of health professionals at medical centers is a significant barrier to adherence. This suggests that medical staff are essential actors in efforts to reduce nonadherence to ART [53]. Furthermore, this study aligns with the perspective of Murungi et al [54], who argued that religious leaders play a crucial role in HIV/AIDS prevention and ART adherence [55].

In addition, our study revealed how viral suppression can be maintained through technology-driven solutions for KPs. Participants identified several tools, including social media, SMS text messages, email, Google Maps, reminder systems on smartphones, artificial intelligence–powered embedded apps, client-focused apps, mobile apps, and interactive systems, as effective means for sustaining viral suppression among these groups. This aligns with Phan et al [56], who demonstrated the potential benefits of digital interventions in promoting adherence to health solutions among people living with HIV/AIDS.

Furthermore, our data revealed that many key demographics are unwilling to disclose their challenges while continuing to engage in unprotected sex. KPs also face a lack of information to help combat virus transmission, as well as insufficient awareness campaigns, bisexual relationships, inadequate knowledge about partners, family and societal pressure to marry at all costs, and self-denial of diagnosis. Several studies corroborate this finding. For instance, Cherutich et al [57] identified a lack of HIV status knowledge as a significant barrier to HIV prevention, care, and treatment activities. In Kenya, high rates of undiagnosed HIV infection are prevalent among gay, bisexual, and other MSM, as well as transgender women [58]. The African Union Commission [59] has demonstrated that high rates of child marriage often coincide with high rates of HIV infection in many countries. Acceptance of HIV status is critical for the effectiveness of HIV tests and related activities, whereas self-denial following a positive diagnosis can hinder adherence [60].

Our findings also identified several significant challenges among stakeholders, including insufficient salary payments, lack of collaboration between stakeholders, lack of motivation, and prevalent biases. Proper stakeholder education and accurate information can positively impact adherence, which supports the conclusions of this research [61].

### Evaluating Relationship With Previous Findings

These findings align with the suggestions of Labrique et al [35], who indicated that the WHO and other international organizations advocate for the use of digital technologies to deliver adherence interventions and suppress viral infections. Previous studies on digital innovations in health care (eg, [22,23,29,62]) demonstrate the availability of various digital solutions. These include real-time medication monitoring and SMS text message reminders, which signal medication events, as well as real-time digital interventions through SMS text message reminders and remote monitoring solutions. The study also confirmed that adolescents are at high risk of transmitting HIV, partly because many individuals with the infection are reluctant to discuss their health openly, thereby contributing to its spread. Additionally, challenges in working with stakeholders include the prevalence of contract employees with unsatisfactory pay, lack of collaboration among stakeholders, a lack of passion for the job, and significant bias. These findings are strongly supported by the study conducted by Ledda et al [62].

### Limitations

This study investigated digital health care interventions for monitoring ARV medications among Nigeria’s KPs through empirical analysis. It is important to acknowledge some limitations, as no research is without flaws. The study focused exclusively on Lagos, and thus, expanding it to include other regions across Nigeria could provide valuable and practical insights applicable to health care facilities nationwide. Furthermore, the study’s sample size was limited to a maximum of 7 key medical actors, and data were collected using an IM approach. Future research should consider conducting mixed methods or strictly quantitative assessments to provide a more comprehensive evaluation.

### Conclusions

The study investigates health professionals’ perceptions of digital health care interventions for monitoring ARV medications among KPs in Nigeria. The study concluded that technology-driven solutions could improve adherence to ART and reduce HIV transmission among KPs. Based on the findings of this study, the following recommendations are made: The government should implement improved policies to encourage positive attitudinal changes among stakeholders, promoting the use of technology-driven solutions to maintain viral suppression among the HIV population in the country; additionally, local governments and international agencies should facilitate awareness campaigns to improve ART sessions among KPs. Strategies should also be developed to help Nigerian health facilities integrate suitable channels for cocreating digital solutions that will increase attendance at ART sessions.

## Abbreviations

ART: antiretroviral therapy
ARV: antiretroviral
HAART: HIV/AIDS antiretroviral therapy
IM: interactive management
KP: key population
LGBTQI: lesbian, gay, bisexual, transgender, queer, and intersex
MSM: men who have sex with men
NGO: nongovernmental organization
TIERs: The Initiative for Equal Rights
WHO: World Health Organization
